# The Rehabilitation of a Patient with Acute Transverse Myelitis After HPV Vaccination—A Case Report

**DOI:** 10.3390/diseases13090281

**Published:** 2025-09-01

**Authors:** Kornelia Kowalik, Piotr Niebrzydowski, Julia Kropidłowska, Alexandra Kvinen, Małgorzata Kusiak-Kaczmarek, Dominika Szalewska

**Affiliations:** 1 Division of Rehabilitation Medicine, Faculty of Health Sciences, Medical University of Gdansk, 80-219 Gdańsk, Poland; 2 Rehabilitation Clinic, University Clinical Center, Aleja Zwycięstwa 30 Street, 80-219 Gdańsk, Poland; pniebrzydowski@uck.gda.pl (P.N.);

**Keywords:** acute transverse myelitis, ATM, human papillomavirus, HPV vaccine, rehabilitation

## Abstract

Acute transverse myelitis (ATM) is a rare, immune-mediated disorder of the spinal cord characterized by sensory, motor, and autonomic dysfunction. Although the human papillomavirus (HPV) vaccine is widely regarded as safe, isolated reports have suggested a potential temporal association with autoimmune neurological events, including ATM. We present a case of a 21-year-old woman who developed ATM two weeks following administration of the first dose of the HPV vaccine (Cervarix). The clinical presentation included rapid-onset paraparesis, sensory deficits, and sphincter dysfunction. An MRI revealed a T2-hyperintense lesion at the Th10–Th12 level. A cerebrospinal fluid analysis showed elevated protein levels. The patient underwent corticosteroid therapy, plasmapheresis, and IVIG, followed by a comprehensive, individualized rehabilitation program. This included balance and stability training, Redcord-based neuromuscular activation, electrostimulation, and pelvic floor therapy. Although no causal link between HPV vaccination and ATM has been established, this case emphasizes the importance of considering post-vaccinal autoimmune phenomena. More importantly, it illustrates the critical role of early, targeted rehabilitation—particularly pelvic floor re-education and neuromodulation—in improving outcomes in patients with significant motor and autonomic deficits.

## 1. Introduction

The human papillomavirus (HPV) vaccine is a key preventive measure against HPV-related diseases. HPV comprises over 200 viruses, with types 16 and 18 responsible for ~71% of cervical cancers and many other anogenital and oropharyngeal malignancies [1]. Routine vaccination, recommended for individuals aged 9 to 45 years, reduces high-risk HPV infections by over 90% when administered before sexual debut [2,3]. The available vaccines (bivalent, quadrivalent, nonavalent) target the main oncogenic types and have been proven to be safe and effective in large clinical trials [4]. Despite this strong safety profile, most adverse reactions are mild and transient [3]. Severe complications are extremely rare, although scattered case reports have described associations with multiple sclerosis, optic neuritis, and Guillain–Barré syndrome [5]. A large meta-analysis by Mouchet et al. found no significant link between HPV vaccination and CNS demyelinating disorders, suggesting such associations may be coincidental or reflect individual predispositions [6]. Acute transverse myelitis (ATM) is a rare but debilitating inflammatory disorder of the spinal cord, presenting with rapidly progressive weakness, sensory disturbances, and autonomic dysfunction [7,8,9]. While no definitive causal link with HPV vaccination has been established, scattered case reports suggest ATM may, in rare cases, occur shortly after immunization. Here, we describe a 21-year-old woman who developed ATM two weeks after receiving her first dose of the bivalent HPV vaccine Cervarix, with subsequent immunotherapy and rehabilitation leading to a partial but significant recovery.

## 2. Case Presentation

A previously healthy 21-year-old female was transferred to the Neurology Ward at the Clinical Center in Poland from a hospital in Slovakia following a diagnosis of acute transverse myelitis. The patient initially presented with neurological symptoms during a recreational hike, experiencing sudden-onset paresthesias in both lower limbs, accompanied by pain in the lumbar spine radiating along the nerve root pathways toward the lower extremities. Within an hour, her condition rapidly deteriorated, leading to difficulty in walking and impaired motor control of the lower limbs.

Upon her initial hospital admission, the clinical examination revealed bilateral paresthesias, paraparesis, dysesthesia in the lower limbs, urinary sphincter dysfunction, and decreased sensation in the anogenital region. The neurological deficits were symmetrical and corresponded to a thoracic spinal cord involvement. Notably, the patient had received the first dose of the HPV vaccine Cervarix approximately two weeks prior to the onset of the symptoms. She denied any recent infections, systemic illnesses, or prior neurological complaints. Her medical history was otherwise unremarkable, and there was no known family history of autoimmune or demyelinating disorders.

The initial diagnostic imaging, including MRI scans of the brain and thoracic and lumbar spine, did not reveal any significant abnormalities. A CSF analysis demonstrated a mild elevation of protein concentration (0.46 g/L) but no pleocytosis, and the neuropanel was negative for common neurotropic viruses. A weakly positive antinuclear antibody (ANA) titer was observed, though its clinical significance remained uncertain at that point. The anti-AQP4 and anti-MOG antibody tests were negative. An empirical treatment with 1 g of intravenous methylprednisolone was initiated and lasted for 5 days under the presumptive diagnosis of an inflammatory myelopathy.

**Figure 1:**
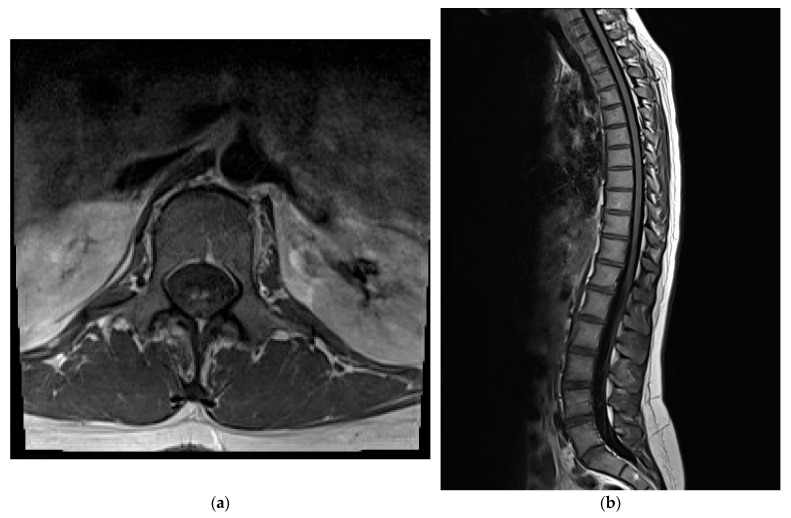
Magnetic Resonance Imaging (MRI) of the spine (T2-weighted). (**a**) Axial view of the spinal cord showing the “snake eye” sign. (**b**) Sagittal view with longitudinal T2-hyperintense lesions from T9 to L2 affecting posterior columns.

A follow-up MRI of the thoracic and lumbosacral spine revealed multiple small T2-hyperintense intramedullary lesions, the most prominent from T9/T10 to T11/T12, involving mainly the posterior spinal cord. At T12/L1 to L1/L2, symmetrical foci were seen in both the anterior and posterior horns, forming the characteristic “snake eye” sign. The post-contrast images showed an enhancement of the cauda equina nerve roots. No compressive lesions or vertebral abnormalities were present. The distribution and intensity of the lesions were stable compared to the prior scans and suggested an inflammatory or autoimmune etiology (Figure 1). Based on the clinical presentation and MRI findings, a diagnosis of acute transverse myelitis was confirmed, and therapeutic plasmapheresis was commenced. The patient underwent a total of five sessions of plasma exchange, resulting in a partial clinical improvement, including a reduction in paresthesias and a gradual return of motor strength.

Further serological testing revealed the presence of IgM antibodies against Borrelia burgdorferi, confirmed by a Western blot analysis. Consequently, she was started on targeted antibiotic therapy with amoxicillin. However, the diagnosis of neuroborreliosis was later excluded due to the absence of inflammatory markers in the CSF. This discrepancy highlighted the diagnostic challenges often encountered in ATM, where incidental findings can mislead the clinical interpretation.

Upon her transfer to the Clinical Center in Poland, the patient’s neurological examination revealed residual motor deficits. The muscle strength in the right lower limb was graded 3/5 in hip flexion and extension, while the left limb showed 4/5 strength. The knee flexion and extension were 4/5 bilaterally, and the ankle movements were preserved at 5/5. The deep tendon reflexes were diminished in the knees and absent at the ankles. A third spinal MRI showed the persistence of the thoracic lesion, consistent with the previous imaging findings. A repeat CSF analysis showed a slightly increased protein level (0.50 g/L) and mild pleocytosis (6 cells/μL). Further blood tests revealed reduced serum vitamin B12 levels and the presence of anti-parietal cell antibodies, indicating a possible autoimmune-mediated gastric pathology.

Due to the previous plasmapheresis treatments, the patient’s IgG and IgA levels were significantly depleted, necessitating an intravenous immunoglobulin (IVIG) supplementation. She received two IVIG infusions; however, during the first administration, she developed an episode of anaphylaxis, requiring an antihistaminic premedication for the continuation of treatment. The therapy was resumed successfully without further complications.

With ongoing physiotherapy at the Neurology Ward, the patient demonstrated progressive improvement in muscle strength and regained partial bladder control. Her motor performance improved to 4/5 strength in hip flexion and extension on the right, 4+/5 on the left, and 5/5 in the knees and ankles. Her sensory improvement remained limited, particularly in the perianal region.

In early September, the patient was transferred to the Rehabilitation Clinic, where she underwent intensive rehabilitation from 1 September to 5 October 2023. On admission, her functional status was assessed using several scales: a modified Rankin Scale (mRS) score of 3, a Barthel Index of 14, and her muscle strength on the Oxford scale was graded at 2. She retained superficial sensation over the labia majora on the right side but lacked anal sensation and had no voluntary sphincter control. The maximal voluntary contraction of the pelvic floor muscles was less than 5 s, and incontinence was present.

**Figure 2:**
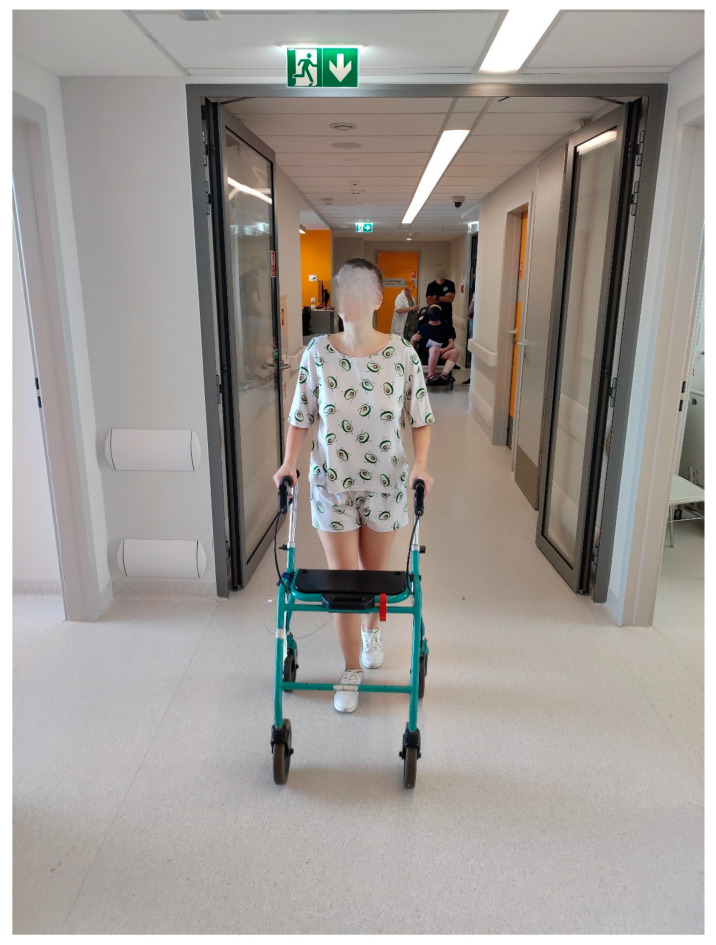
The patient during gait training using a walker.

Her rehabilitation program included a comprehensive regimen: daily 60 min sessions involving breathing exercises, low-resistance active movement, conditioning and isometric training, balance re-education, and stair-climbing drills along with assisted ambulation with a walking aid (Figure 2). By mid-September, she began exercising with the Redcord suspension system, which allows for neuromuscular re-education and stabilization under reduced weight-bearing conditions. She also trained on stabilometric platforms using biofeedback technology to improve her postural control and proprioception. In response to the hypersensitivity in the lower limbs, the patient received massage as a part of a desensitization therapy aimed at modulating the abnormal sensory input.

Electrostimulation of the pelvic floor muscles was performed every two days, complemented by kinesiotherapy focusing on core stability. Following the third electrostimulation session, the patient reported the return of minimal sensory perception and discomfort with pressure stimuli. After the fifth session, she noticed subtle sensations in the anal region, indicating early neurological recovery.

By the time of her discharge from the Rehabilitation Clinic, she had shown significant functional gains: improved voluntary control of bladder function, increased strength of pelvic floor contractions (sustained for 15 s), and a marginal enhancement of superficial sensation. She still experienced occasional urinary incontinence, sometimes accompanied by defecation, but no longer required assistance with daily activities. Her mRS improved to 1 and her Barthel Index to 20. She was advised to continue pelvic floor training at home using electrostimulation equipment, with a focus on the ischial region, and she was educated about strategies to assist defecation and urination.

During an oral follow-up consultation conducted 18 months post-discharge, the patient reported ongoing care under multiple out-patient clinics, including neurology, gastroenterology, urology, immunology, and rheumatology. She continues to experience intermittent neuropathic pain in the lower extremities, for which she attends private physiotherapy sessions. Additionally, she reports persistent constipation and occasional gas incontinence, accompanied by reduced urethral sphincter tone, necessitating the use of sanitary pads. Despite these residual symptoms, the patient subjectively rates her overall condition as very good. Her modified Rankin Scale (mRS) score was 1, and her Barthel Index showed marked improvement (Table 1).

## 3. Discussion

The presented case exemplifies a rare but serious neurological complication—acute transverse myelitis—potentially triggered by immunological mechanisms following HPV vaccination. Although causality cannot be firmly established, the temporal relationship, the absence of a prior neurological disease, and the exclusion of alternative etiologies provide a sufficient basis for discussing this case in the context of vaccine safety monitoring.

ATM is a neuroinflammatory disorder characterized by focal spinal cord inflammation, resulting in sensory, motor, and autonomic dysfunction. While it can occur in the context of systemic autoimmune diseases such as systemic lupus erythematosus or Sjögren’s syndrome, it is often post-infectious or idiopathic [10]. The precise pathogenesis of ATM remains incompletely understood, but the current hypotheses emphasize molecular mimicry, bystander activation, and dysregulated immune responses. Following vaccination or viral infection, host antigens may be mistakenly targeted by autoreactive T-cells due to shared epitopes, resulting in inflammation and demyelination within the spinal cord [11].

Although the HPV vaccine has been rigorously tested and found to be safe in millions of individuals worldwide, isolated case reports have documented immune-mediated neurological syndromes post-vaccination. In 2015, Baker et al. reviewed multiple such cases, including myelitis, and hypothesized the involvement of vaccine adjuvants in some autoimmune phenomena. Cervarix, specifically, contains the AS04 adjuvant—a combination of aluminum hydroxide and monophosphoryl lipid A—which enhances the immune response by stimulating Toll-like receptor 4 (TLR4). While this adjuvant has been approved and used extensively, some studies have suggested it may contribute to rare immune dysregulation in predisposed individuals [12].

In the current case, the onset of neurological symptoms occurred approximately 14 days after the administration of the vaccine, a window commonly associated with immune-mediated reactions.

While coincidental infections—such as asymptomatic viral illnesses—could not be completely ruled out, the extensive microbiological workup, including CSF PCR, was negative for a wide array of pathogens. The transient positivity for Borrelia burgdorferi IgM, which led to the initial antibiotic treatment, was eventually interpreted as a false positive or incidental exposure, particularly as no supporting CSF findings were present.

The presence of anti-parietal cell antibodies and vitamin B12 deficiency suggests an underlying autoimmune gastritis, which raises the possibility of a broader predisposition to autoimmune reactions [13]. Such a “primed” immune environment may theoretically have facilitated a pathological response to the vaccine-induced activation of the dendritic cells and T-helper lymphocytes, although no specific autoimmune disease was diagnosed at that time and the weakly positive ANA remained of unclear significance. Interestingly, a comparable case has been reported of a patient with parietal cell antibody-positive gastritis who developed acute transverse myelitis within the context of autoimmune polyglandular syndrome type 3B, requiring an aggressive immunosuppressive therapy and leaving residual paraparesis [14]. Although our patient does not fulfill the criteria for such a syndrome, both of the cases highlight that immune predisposition may underlie neurological complications; however, these remain isolated observations and cannot establish causality.

The diagnosis of ATM was ultimately supported by the patient’s clinical profile, MRI findings, and a partial response to the immunomodulatory therapies. The longitudinally extensive lesion involving the posterior columns of the thoracic cord, observed in the T10–T12 region, correlated well with her sensory and motor deficits. It is worth noting that posterior column involvement typically results in proprioceptive loss and gait ataxia, which were among the patient’s initial symptoms. The absence of brain lesions on the MRI supported the exclusion of multiple sclerosis (MS), while the lack of oligoclonal bands in the CSF and negative anti-AQP4 and anti-MOG antibody results argued against neuromyelitis optica spectrum disorder (NMOSD) [8].

The management of ATM generally consists of high-dose corticosteroids to reduce inflammation, followed by plasmapheresis or IVIG in steroid-refractory cases [15]. In this instance, the patient received a prompt steroid therapy and underwent a plasma exchange with modest benefit. Due to her hypogammaglobulinemia following plasmapheresis, she also required IVIG replacement therapy [16]. Although she experienced anaphylaxis during the first infusion, this was successfully managed, and the subsequent administrations were tolerated.

To contextualize our case and underscore its novelty, it is valuable to compare it with the previously reported instances of ATM following HPV vaccination. Fernández-Fournier et al. (2014) described a 14-year-old girl who developed cervical ATM just 3 days after receiving the quadrivalent HPV vaccine (qHPV)—the shortest latency reported in the literature [17]. In contrast, Badarny et al. (2020) reported a hemorrhagic form of ATM emerging approximately one month post-vaccination, adding a rare radiological subtype to the spectrum [18]. Earlier safety reviews, including Stratton et al. (2011/2012), have cited ATM cases following HPV vaccination, although without detailed clinical descriptions [19] (Table 2).

Our case adds a distinct perspective by documenting a 21-year-old woman who developed ATM two weeks after the first dose of the bivalent HPV vaccine (Cervarix), characterized by thoracic cord involvement (T9–T12), marked motor and sphincter dysfunction, and partial neurological recovery after multimodal immunotherapy combined with intensive rehabilitation.

Taken together, the available evidence highlights the significant variability in latency (ranging from days to weeks), lesion characteristics (cervical, thoracic, hemorrhagic), and outcomes (steroid-responsive vs. rehabilitation-dependent recovery). This comparison strengthens the argument for continued vigilance and detailed reporting of such rare post-vaccination events while also underscoring the importance of individualized rehabilitation strategies in optimizing recovery.

Rehabilitation played a pivotal role in the patient’s recovery. ATM often leaves patients with persistent deficits, especially in sphincter control and proprioception, which require long-term management. An intensive physiotherapy focusing on neuroplasticity, balance, strength, and motor control was crucial in her functional improvement. The use of the Redcord system and stabilometric platforms reflects the modern rehabilitation strategies aimed at engaging both the central and peripheral pathways for sensory and motor retraining [20].

Pelvic floor muscle electrostimulation was included as a supportive element of the rehabilitation program and appeared to contribute to the gradual improvement of her perineal sensation and the partial recovery of her bladder control. In the context of acute transverse myelitis (ATM), where autonomic dysfunction—including neurogenic bladder—is frequently significant and recovery may be incomplete, the early use of pelvic floor-targeted interventions is considered beneficial.

Although randomized clinical studies, such as the one conducted by Elmelund et al., did not demonstrate a clear superiority of intravaginal electrical stimulation (IVES) over pelvic floor muscle training (PFMT) alone in women with incomplete spinal cord injury, both of the approaches were associated with meaningful improvements in urinary continence and quality of life [21]. This suggests that neuromuscular stimulation may serve as a useful adjunct to conventional therapy, particularly in patients who initially exhibit limited voluntary activation of the pelvic floor musculature.

In the present case, the marked improvement in both the Barthel Index and modified Rankin Scale scores over the course of one month supports the effectiveness of a multimodal, individualized rehabilitation approach in facilitating functional recovery, even in the presence of severe initial deficits.

Despite these advances, the patient’s recovery remained incomplete at the time of discharge, particularly in terms of fine sensory discrimination and sphincter control. A long-term follow-up and continued rehabilitation are necessary in such cases. Additionally, given the suspected autoimmune background (i.e., the presence of anti-parietal cell antibodies and B12 deficiency), a further gastroenterological and immunological workup may be warranted to identify latent autoimmune gastritis or pernicious anemia.

At the 18-month follow-up, the patient demonstrated good overall functional recovery and subjectively rated her health as very good, despite her residual symptoms such as neuropathic pain, constipation, and reduced urethral sphincter tone. She remains under the care of several outpatient specialists and does not currently engage in sports activities. While her general condition is favorable, the ongoing need for multidisciplinary follow-up, the limitations in daily functioning (e.g., during long journeys), and the persistent symptoms may carry a potential psychological burden, which should also be acknowledged when assessing long-term quality of life in such cases.

The rarity of ATM post-vaccination presents a unique challenge: how to balance individual case observations against the overwhelming public health benefits of vaccination. It is essential to acknowledge that sporadic adverse events may occur, but causality is difficult to establish without systematic data. Surveillance systems such as VAERS (Vaccine Adverse Event Reporting System) in the US and EudraVigilance in the EU are critical tools in identifying signals, but they rely heavily on clinician reporting and cannot determine causation on their own. Large-scale, controlled studies are needed to better understand the risk profile for such complications, especially in genetically or immunologically predisposed individuals.

Both at the Slovak facility and during hospitalization in the neurology department, a possible causal link between HPV vaccination and ATM was considered. The main argument in favor of such a link was the close temporal relationship between the vaccination and the symptom onset, along with the absence of other potential triggers (e.g., Lyme disease was excluded during diagnostics). Nevertheless, a single case report does not allow conclusions about causality. In light of Bradford Hill’s criteria (such as strength and consistency of association, biological plausibility, and coherence with epidemiological evidence), our observations should be regarded as a signal warranting further investigation rather than proof of a causal relationship [22].

While this case contributes to the body of anecdotal evidence suggesting a possible link between HPV vaccination and ATM, it should not deter the implementation of HPV immunization programs. Rather, it underscores the importance of informed consent, patient education, and close monitoring of post-vaccination symptoms. Patients with a personal or family history of autoimmune diseases might benefit from individualized counseling before vaccination, though no current guidelines recommend a routine pre-vaccination screening for autoimmunity.

In follow-up correspondence, the patient disclosed that she had not received additional doses of the HPV vaccine following the onset of her neurological symptoms. While the standard Cervarix vaccination schedule comprises three doses, the emerging evidence supports the efficacy of a single-dose regimen. A study published by Fokom-Defo et al. demonstrated that a single dose of the HPV vaccine provides comparable protection against HPV types 16 and 18 as the traditional three-dose schedule [23]. Therefore, even if the patient chooses not to proceed with the remaining doses—whether due to clinical considerations or personal preference—there remains a substantial likelihood that she retains effective immunological protection against the most oncogenic strains of HPV [24,25].

## 4. Conclusions

This case report describes a rare instance of acute transverse myelitis in a young woman following the administration of the first dose of the bivalent HPV vaccine Cervarix. Although causality cannot be conclusively proven, the temporal association and the absence of alternative explanations support the hypothesis of an immune-mediated mechanism potentially triggered by vaccination. The clinical presentation, diagnostic approach, and therapeutic interventions followed the standard neurological and rehabilitative protocols, resulting in a partial but significant recovery.

Importantly, this report highlights the necessity of vigilance in recognizing and managing rare post-vaccination complications without undermining the fundamental importance of HPV vaccination in cancer prevention. Further research is needed to elucidate the immunopathogenic mechanisms that may predispose certain individuals to such adverse events. Meanwhile, this case reinforces the critical role of comprehensive rehabilitation in restoring function and improving quality of life after severe spinal cord inflammation.

In conclusion, while HPV vaccines remain a cornerstone of the public health strategy against HPV-related cancers, healthcare providers should be aware of rare but serious neurological adverse events. A timely recognition, a thorough diagnostic workup, and a multidisciplinary approach to management—including immunotherapy and intensive rehabilitation—can significantly influence the outcomes. The continued reporting and analysis of such cases are essential to refining our understanding of vaccine safety and individual susceptibility to adverse reactions.

## Figures and Tables

**Table 1:** The assessment of the patient’s condition before and after rehabilitation and 18 months post-discharge.

Prior to Rehabilitation	After One Month of Rehabilitation	18 Months Post-Discharge
mRS * = 3	mRS * = 1	mRS * = 1
Barthel Index = 14	Barthel Index = 20	Barthel Index = 95
muscle strength (Oxford scale) = 2	muscle strength (Oxford scale) = 3	muscle strength (Oxford scale) = 4
sphincter control = involuntary	sphincter control = partial, improved	sphincter control = substantially recovered
maximal pelvic floor contraction < 5 s	maximal pelvic floor contraction = 15 s	maximal pelvic floor contraction > 15 s

* mRS = modified Rankin Scale

**Table 2:** Case reports of ATM after HPV vaccination.

Author	Patient Details	Timing Post-Vaccination	Presentation Type	Key Features and Outcome
Our Case Report	21-year-old woman	2 weeks after Cervarix	Thoracic ATM	T9–T12 lesion, paraparesis, sphincter dysfunction; treated with steroids, plasmapheresis, IVIG + intensive rehab; marked improvement
Fernández-Fournier et al., 2014 [17]	14-year-old girl	3 days after qHPV	Cervical ATM	MRI C1–C2 lesion, CSF OCB; improved post-steroids
Badarny et al., 2020 [18]	30-year-old woman	~1 month after HPV	Hemorrhagic ATM	MRI with intramedullary hemorrhage; treated with steroids; marked improvement
Stratton et al., 2011/2012 [19]	Cases cited in safety review	Variable	(unspecified)	Included in systematic vaccine safety analysis

## Data Availability

The data can be obtained from this article.

## References

[1] European Centre for Disease Prevention and Control (2019). Efficacy, Effectiveness and Safety of HPV Vaccination in Women Undergoing Conisation: Systematic Review.

[2] Sharpless K.E., Marcus J.Z., Kuroki L.M., Wiser A.L., Flowers L. (2023). ASCCP Committee Opinion: Adjuvant Human Papillomavirus Vaccine for Patients Undergoing Treatment for Cervical Intraepithelial Neoplasia. J. Low. Genit. Tract Dis..

[3] Cheng L., Wang Y., Du J. (2020). Human Papillomavirus Vaccines: An Updated Review. Vaccines.

[4] American College of Obstetricians and Gynecologists (2020). Human papillomavirus vaccination: ACOG Committee Opinion No. 809. Obstet. Gynecol..

[5] Boender T.S., Bartmeyer B., Coole L., Wichmann O., Harder T. (2022). Risk of Guillain–Barré syndrome after vaccination against human papillomavirus: A systematic review and meta-analysis, 1 January 2000 to 4 April 2020. Euro Surveill..

[6] Mouchet J., Salvo F., Raschi E., Poluzzi E., Antonazzo I.C., De Ponti F., Bégaud B. (2018). Human papillomavirus vaccine and demyelinating diseases—A systematic review and meta-analysis. Pharmacol. Res..

[7] Fiszer U. (2006). Ostre poprzeczne zapalenie rdzenia kręgowego. Pol. Prz. Neurol..

[8] Simone C.G., Emmady P.D. (2025). Transverse Myelitis. StatPearls [Internet].

[9] West T.W. (2013). Transverse myelitis—A review of the presentation, diagnosis, and initial management. Discov. Med..

[10] Tristano A.G. (2009). Autoimmune diseases associated with transverse myelitis. Review. Invest. Clin..

[11] Naeem F.N., Hasan S.F.S., Ram M.D., Waseem S., Ahmed S.H., Shaikh T.G. (2022). The association between SARS-CoV-2 vaccines and transverse myelitis: A review. Ann. Med. Surg..

[12] Baker B., Guimarães L.E., Tomljenovic L., Agmon-Levin N., Shoenfeld Y. (2015). The safety of human papilloma virus-blockers and the risk of triggering autoimmune diseases. Expert Opin. Drug Saf..

[13] Rodríguez-Castro K.I., Franceschi M., Noto A., Miraglia C., Nouvenne A., Leandro G., Tiziana M., Gian L.d.A., Francesco D.M. (2018). Autoimmune diseases in autoimmune atrophic gastritis. Acta Biomed..

[14] Vences M.A., Morocho-Pinedo M., Oliveros-Ramirez R.J., Ballena-Cupe L.C., Alvarez-Márquez J.C., Villafuerte-Espinoza M., Urrunaga-Pastor D. (2023). Transverse myelitis associated with autoimmune polyglandular syndrome type 3B: A case report in Peru. Neurol. Argent..

[15] Greenberg B.M. (2011). Treatment of acute transverse myelitis and its early complications. Continuum.

[16] Compagno N., Malipiero G., Cinetto F., Agostini C. (2014). Immunoglobulin replacement therapy in secondary hypogammaglobulinemia. Front. Immunol..

[17] Fernández-Fournier M., Díaz de Terán J., Tallón Barranco A., Puertas I. (2014). Early cervical myelitis after human papilloma virus vaccination. Neurol. Neuroimmunol. Neuroinflamm..

[18] Badarny S., Badarny Y., Goldfeld M., Wakid H. (2020). Hemorrhagic Myelitis after Papilloma Virus (HPV) Vaccination. Austin J. Mult. Scler. Neuroimmunol..

[19] National Academies of Sciences, Engineering, and Medicine (2011). Human papillomavirus vaccine safety. Adverse Effects of Vaccines: Evidence and Causality.

[20] Sadowsky C.L., Becker D., Bosques G., Dean J.M., McDonald J.W., Recio A., Frohman E.M. (2011). Rehabilitation in transverse myelitis. Continuum.

[21] Elmelund M., Biering-Sørensen F., Due U., Klarskov N. (2018). The effect of pelvic floor muscle training and intravaginal electrical stimulation on urinary incontinence in women with incomplete spinal cord injury: An investigator-blinded parallel randomized clinical trial. Int. Urogynecology J..

[22] Shimonovich M., Pearce N., Cheung K.L., Saracci R., Porta M. (2021). Assessing causality in epidemiology: Revisiting Bradford Hill to incorporate developments in causal thinking. Eur. J. Epidemiol..

[23] Fokom-Defo V., Dille I., Fokom-Domgue J. (2024). Single dose HPV vaccine in achieving global cervical cancer elimination. Lancet Glob. Health.

[24] Stanley M., Schuind A., Muralidharan K.K., Guillaume D., Willens V., Borda H., Jurgensmeyer M., Limaye R. (2024). Evidence for an HPV one-dose schedule. Vaccine.

[25] Malvi S.G., Esmy P.O., Muwonge R., Joshi S., Poli U.R.R., Lucas E., Verma Y., Lucksom P.G., Shah A., Patel B. (2023). A prospective cohort study comparing the efficacy of 1 dose of quadrivalent HPV vaccine to 2 and 3 doses at an average follow-up of 12 years post-vaccination. IARC News.

